# Evaluation of sexual attitude and sexual function in menopausal age; a population based cross-sectional study

**Published:** 2013-08

**Authors:** Somayeh Hashemi, Fahimeh Ramezani Tehrani, Masumeh Simbar, Mehrandokht Abedini, Hamid Bahreinian, Roya Gholami

**Affiliations:** 1 *Reproductive Endocrinology Research Center, Research Institute for Endocrine Sciences, Shahid Beheshti University of Medical Sciences, Tehran, Iran.*; 2 *Department of Reproductive Health, Shahid Beheshti University of Medical Sciences, Tehran, Iran. *; 3 *Department of Geriatric Health Care Department, Deputy of Health, Ministry of Health and Medical Education, Tehran, Iran.*

**Keywords:** *Attitude*, *Sexualbehavior*, *Menopause*

## Abstract

**Background:** Menopause and its physical, hormonal and psychosocial changes could affect women’s sexual function. There are controversial results regarding relationship between sexual attitudes and function.

**Objective:** We aimed to evaluate sexual attitudes and sexual function among Iranian menopausal age women.

**Materials and Methods:** This population based cross-sectional study was carried out on 225 menopausal women, aged 45-65 years. Based on a self-made questionnaire data were collected about women’s socio-demographic characteristics, attitudes regarding sexuality and sexual function. Data were analyzed using SPSS and sexual function was compared between three groups of women who had positive, medium and negative attitudes regarding sexuality.

**Results:** The mean age of women was 53.11±4.56 years. Seventy percent of them had at least one sexual problem. Feeling of dyspareunia was significantly different between three categories of attitudes regarding sexuality (p=0.03). Comparing data obtained on their attitudes, sexual desire, orgasm and dyspareunia demonstrated significant differences (p=0.03, 0.04, and 0.04 respectively).

**Conclusion:** Attitude regarding sexual function has a great impact on sexual activity of postmenopausal women that need to be considered in their health care programming.

## Introduction

Menopause is a course of life identified by hormonal changes along with important social alterations. Improving health services and quality of life of middle-aged and elderly women, increases life expectancy. Women commonly spend one-third of their lives during menopause (1). Menopause is characterized by hormonal, physical and pathological changes which could affect sexual function adversely (2). 

The postmenopausal ovary continues to secrete a substantial amount of androgens. It has been known for three decades that the climacteric ovary contributes 50% of testosterone and 30% of androstenedione to the circulation. Although this process seems to increase sexual desire, lower estrogen levels in menopausal women may lead to decreased vascular engorgement and vaginal secretions during sex, resulting in a lower feeling of arousal and a disruption in the sexual relationship (3, 4). Sexual activity plays an important role in menopause and has tremendous effects on women’s health, well-being, self-confidence and their quality of life (5-8). Besides the mentioned items, psychological, mental and behavioral changes that usually occur during menopause also affect women’s sexual activity (2, 9).

It is commonly assumed that natural menopause contributes dramatically to a decline in sexual activity (10). During menopause, declining oestrogen level is one of the main causes of sexual dysfunction, but on the other hand increasing free testosterone affects the neurotransmitter systems involved in sexual behavior positively, since serum testosterone level is directly correlated with sexual desire and sexual behavior (11, 12). Although sexual function during menopause is largely influenced by biologic changes, women’s ethnic background and socio-cultural factors also play a key role in levels of their sexual activity (13, 14). In many societies, cultural notions and experiences impact a woman’s perception of menopause and sexual function in this period (7, 15). 

It seems that in addition to biological and socio-cultural status, the attitudes of menopausal women regarding sexuality also affect their sexual function. There are limited studies that have focused on sexual attitudes and sexual function or behavior among menopausal women and they report diverse results. Wang *et al* reported that sexual activity is related to sexual attitudes and knowledge among men and women aged over 65years (16). Although it is a fact that sexual attitudes of older women are restricted, studies didn't report any age-related decrease in sexual activity, they also didn't confirm a decline in frequency of sexual intercourse among menopausal women (17, 18). 

The lack of population based studies has confined generalization of results of previous studies. Moreover since cultural obstacles also restrict sexual activity of menopausal women in Iran, and there is no similar study of societies with cultural problems similar to Iranian women, we designed this population based study to evaluate sexual attitudes and sexual function among Iranian menopausal age women (5). 

## Materials and methods

The present study is a population based cross-sectional study of 225 women, aged 45-65 years, conducted during 2009-2010. This study was conducted along with the national population-based study of prevalence of morbidities among reproductive age women. The women eligible were recruited among women living in urban areas of four randomly selected provinces in different geographic regions of Iran, i.e. Ghazvin (Central), Kermanshah (East), Golestan (North) and Hormozgan (South). A stratified, multistage probability cluster sampling method was used, each cluster comprising of seven households. 

The frame for the selection of the sampling units was based on the Iranian household lists available in the Health Department and the cluster was selected systematically. The proportion of required samples in each province was calculated based on the total number of women aged 45-65 years living in the urban areas of each of these provinces. All women who were married, lived with their spouse, were naturally menopause, were psychologically normal and didn’t use drugs for psychological problems and had no history of hysterectomy or oophorectomy were included. Women who were under hormone replacement therapy were excluded from study. The dropout rate was below 5 percent. Written informed consent was obtained from all participants before interviews. This research was approved by Ethics Committee of Research Institute of Endocrine Sciences, Shahid Beheshti Medical Sciences University. It notes worthy that this research was funded in the form of Paragraph "d" project of Article 145 of the Fourth Development Plan of Iran and commissioned by the Family Health Department of Health Deputy of Ministry of Health and Medical Education.

Three questionnaires were completed by all participants, i.e. demographic information, sexual attitudes and the sexual function questionnaires. Sexual attitudes self-made questionnaire included 12 questions that required respondents to indicate whether they agree or disagree with the statements using the following scales: 1- disagree, 2- undecided, 3- agree, 4- strongly agree. A final score was obtained for the total scale by summing responses graded with scores, ranging between 12-48. Lower scores represented negative sexual attitudes while higher scores showed positive attitudes. Sexual attitudes scores of participants ranged between18-48. They were categorized as tertiles; negative (scores 17-32), medium (scores 33-38) and positive (scores 39-48). Sexual function self-made questionnaire included 15 questions. Questions were intended to evaluate various aspects of sexual function including desire, satisfaction, lubrication, orgasm, dyspareunia and frequency of sexual activity during a month.

This self-designed questionnaire including questions on socio-demographic characteristics, sexual attitudes and sexual function. Its face and content validity were evaluated by 15 gynecologists and psychiatrists who are expert in the field of sexual health. We changed the questionnaires based on their comments. The reliability of the questionnaire was assessed using test-retest and inter-rater method. In these methods, first a trained questioner completed questionnaires for 30 participants, then another observer filled the same questionnaire. After one week the first questioner completed them again. Using statistical analysis we compared results of two observatories as inter-rater reliability and two assessment of first questioner as test-retest reliability. Both confirmed by r=0.91 and r=0.85 respectively.

**Statistical analysis**

Continuous variables were checked for normality, using the one-sample Kolmogorov-Smirnoff test, and expressed as mean±SD and/or median (IQ 25-75), as appropriate. Frequency of sexual relations during one month was compared using t-test between groups of sexual attitudes. The nominal and categorical variables, expressed as percentages, were compared using Pearson’s X test. 

## Results

Participants had a mean age of 53.11±4.56 years. Approximately sixty eight percent had been living for over 30 years with their spouses. The mean age at menopause and SD was 47.35±6.7 years, as presented in table I, the mean age of women differed significantly between women with different sexual attitudes (p=0.018). Seventy percent of women expressed at least one sexual problem in terms of desire (46.3%), lubrication (25%), orgasm (27%) and satisfaction (21.3. Based on our results 17.8% of menopausal women often or always experienced dyspareunia. Prevalence of changes in sexual desire and frequency of sexual activity after menopause based on the women’s sexual attitudes are shown in table II. 

**Figure 1:**
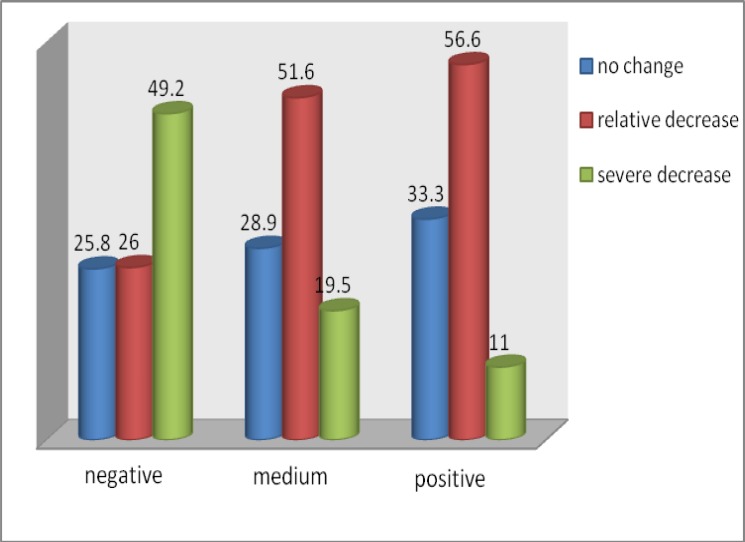
Prevalence (%) of severity of changes in sexual desire among menopausal women, based on their sexual attitudes

Results have shown that changes in sexual desire in women with various sexual attitudes differ significantly (p=0.005); however no association between changes in frequency of sexual activity and women’s sexual attitudes was observed (p=0.191). Severity of changes in sexual desire after menopause in women with negative, medium or positive sexual attitudes is illustrated in figure1. Prevalence of levels of women’s sexual function, based on their attitudes regarding sexual activities is shown in table III. As given in table III, prevalence of orgasm differs significantly between the two groups of negative and positive sexual attitudes (p=0.044). Feeling of dyspareunia shows also significant differences between the three groups of sexual attitudes or the two groups of negative and positive attitudes (p=0.033 and p=0.049 respectively). Results have shown that prevalence of low sexual desire in women with negative sexual attitudes is significantly higher than moderate and positive sexual attitudes (18.6, 11.3 and 6.6% respectively). 

Comparing sexual satisfaction and sexual lubrication, our findings reported that there are no significant difference either between the three groups of sexual attitudes (p=0.17 and 0.74 respectively) or between the two groups of negative and positive attitudes (p=0.08 and 0.43).

**Table I:** Characteristics of menopausal women, based on their sexual attitudes

		**Sexual attitude**			**p-value**
		**Positive (n=66)**	**Medium (n=63)**	**Negative (n=59)**	
Age (mean ± SD) (y)		54.5 ± 4.6	53.0 ± 3.8	52.07 ± 5.3	0.018*
Age difference with husband (mean ± SD) (y)		4.06 ± 7.4	5.1 ± 5.6	8.2 ± 4.7	0.001*
Duration of marriage (mean ± SD)		34.3 ± 7.4	33.7 ± 7.05	33.8 ± 8.7	0.904*
Duration of menopause (mean ± SD)		6.06 ± 5.4	5.28 ± 6	6.57 ± 7.8	0.592*
Husband’s sexual impotency (%)					0.422**
	Yes	3	6	9	
	No	97	94	91	

p<0.05 was considered as significant.

* Data were analysis between three groups using ANOVA.

** Data were analysis between three groups using Chi-square.

**Table II:** Prevalence of alteration in sexual desire and activity in menopausal women based on their sexual attitudes

**Alteration after menopause**		**Sexual attitude**			**Statistical analysis**
		**Negative (n=59)**	**Medium (n=63)**	**Positive (n=66)**	
Sexual desire					
	No change (%)	25	28.9	33.3	p_1_:0.001 p_2_:0.005
	Moderate decrease	25.8	51.6	56.6	
	Severe decrease	49.2	19.5	11	
Frequency of sexual activity (%)					
	No change	34.4	32.6	30	p_1_:0.081 p_2_:0.191
	Moderate decrease	34.4	42.1	58.1	
	Severe decrease	31.2	25.3	11.9	

P_1_: Analyses were done between 2 groups of negative and positive sexual attitudes.

P_1_: Analyses were done between 3 groups of negative, medium and positive sexual attitudes.

p<0.05 was considered as significant. Percentages were compared between two or three groups using Chi-square analysis.

**Table III:** Prevalence of menopausal women’s sexual function based on their sexual attitudes

**Sexual function**		**Sexual attitude**			**Statistical analysis**
		**Negative (n=59)**	**Medium (n=63)**	**Positive (n=66)**	
Desire (%)					
	High	5.9	10.4	5.1	p_1_:0.033 p_2_:0.064
	Moderate	75.5	78.2	88.3	
	Low or none	18.6	11.3	6.6	
Satisfaction (%)					
	High	9.4	10	20.2	p_1_:0.081 p_2_:0.172
	Moderate	30	45.7	45.5	
	Low or none	60.6	44.3	36.3	
Lubrication (%)					
	Often or always	18.9	26.6	35	p_1_:0.43 p_2_:0.74
	Sometimes	53.7	45.4	39.3	
	Rarely or never	27.4	28	25.7	
Orgasm (%)					
	Often or always	16	33.8	27.2	p_1_:0.044 p_2_:0.094
	Sometimes	29.1	33.8	53.7	
	Rarely or never	54.9	32.4	19.5	
Dyspareunia (%)					
	Often or always	18.1	20.5	10.6	p_1_:0.049 p_2_:0.033
	Sometimes	33.7	22	39.4	
	Rarely or never	39.2	57.5	51	

P_1_: Analyses were done between 2 groups of negative and positive sexual attitudes.

P_1_: Analyses were done between 3 groups of negative, medium and positive sexual attitudes.

p<0.05 was considered as significant. Percentages were compared between two or three groups using Chi-square analysis

## Discussion

Various aspects of women’s life were influenced by menopause. Transition to menopause is associated with many biological, psychological and social changes have great impacts on their sexual function   (11, 19) . In this study we found that two thirds of menopausal women suffered from at least one sexual problem. Sexual desire and orgasm were the two most common affected domains among our participants. Although the effects of menopause on health or sexuality is still not clear, studies indicate a significant decrease in menopausal women’s desire, arousal, orgasm and frequency of sexual activity (10, 20, 21). Sexual function could be affected by factors such as the prior level of sexual function, change in partner status, feelings for a partner, oestradiol levels and female identity and culturally dependent lifestyle (22-24). 

Results from various studies differ. In a survey conducted in six European countries on 1805 postmenopausal women, aged 50-60 years, approximately one-third reported that they experienced a reduced sex drive, whereas 53% had become less interested in sex (19). Results of a study conducted in South America on 534 women aged 40-64 years showed that 51.3% presented with at least one sexual complaint (low sexual desire 37.8%; arousal disorder 33.6%; dyspareunia 34.8%; and orgasmic disorder 26%) (25). De Lorenzi and Saciloto reported 60.6% decrease in sexual desire in menopausal women   (26) . Findings of a study conducted on Iranian menopausal women showed that 70.3% of subjects reported a decrease in sexual activity after menopause and 56.4% of subjects had sexual dysfunction. Frequencies of dyspareunia and decreased sexual desire were 55.6 and 70% respectively   (5) . 

According to our results, negative sexual attitudes have adverse effect on women’s sexual desire and orgasm. We also found a significant association between sexual attitudes and experiencing dyspareunia. Evaluating the association between sexual attitudes and changes in sexual desire and frequency of sexual activity after menopause, we found that negative attitudes toward sex are associated with severe decrease in sexual desire; however frequency of sexual activity in menopausal women is independent from sexual attitudes. As a result it seems that improving sexual attitudes of late reproductive aged women could enhance their sexual activity in both desire and sexual function during menopausal age. Some studies indicate that menopausal transition potentially impact sexual attitudes and behavior (27, 28). 

Nappi *et al* also showed greater sexual problems following menopause, when women often suffer from psychological symptoms. In contrast Dennerstein *et al* found no significant change in positive or negative mood scores; however they did observe striking changes in female sexual function during the menopausal transition (10, 29-31). While one study reports that the changes accompanied with menopause affect mood scores negatively, no direct relationship between mood scores and hormone levels has been documented (31). Conversely evidence suggests that androgen affects mood and sexual function; these studies showed striking positive effects of testosterone on mood and on sexual function of middle-aged women (32, 33). 

Avis *et al* believe that transition to menopause has fewer effects on sexual function than relationship variables, cultural environment and attitudes regarding sex (13). The main strength of this study is its methodology. Our study is a community based study with a high response rate (95%), unlikely to be influenced by sample selecting biases; it facilitates the eventuality to generalize results to menopausal Iranian women. Furthermore we have evaluated both attitudes regarding sexuality and sexual function. However we have the limitation of not using daily record for assessing sexual function.

## Conclusion

In conclusion we found more problems in sexual desire and orgasm among menopausal women with negative sexual attitudes. However frequency of sexual activity didn't show significant differences between menopausal women with various sexual attitudes. Considering current increase in life expectancy, women have longer menopausal duration. Health providers in this field can enhance women’s understandings and sexual attitudes; facilitate marital relationship during this particular stage.

## Conflict of interest

We certify that there is no conflict of interest with any financial organization regarding the material discussed in the manuscript.
